# Motivated to Gain: Awareness of an Impending Ending and the Ending Effect

**DOI:** 10.3389/fpsyg.2018.02717

**Published:** 2019-01-17

**Authors:** Cai Xing, Yuqi Meng, Derek M. Isaacowitz, Yunqiang Song, Jiajie Cai

**Affiliations:** ^1^Department of Psychology, Center for Social Psychology and Brain Sciences, Renmin University of China, Beijing, China; ^2^Department of Psychology, Northeastern University, Boston, MA, United States; ^3^Department of Psychology, Renmin University of China, Beijing, China

**Keywords:** ending effect, motivational shift, risky decision-making, Socioemotional Selectivity Theory, visual reaction time task

## Abstract

The ending effect describes the phenomenon that individuals are more risk-taking during the final round of a series of risky decision tasks. Previous research suggests that the ending effect might be caused by a motivational shift induced by changes in time perception. However, none of the existing research directly tested the motivational state immediately before the last round of a series of risky decision tasks. To fill in this gap of knowledge, the present study tested whether this motivational shift indeed occurs immediately before the last round. All participants worked on 11 rounds of risky decision tasks, half of them knew that the decision tasks included 11 rounds, whereas the other half did not know. Before the last round of the risky decision tasks, all participants completed a visual reaction time task. It was found that, compared with participants who were not aware of the impending ending, those who knew they were approaching the last round responded to peripherally located character strings appearing immediately after gain-related words slower than those appearing after loss-related words, suggesting that perceived endings lead participants to be more motivated toward gaining rewards. This work provides critical evidence which supports the motivational account of the ending effect proposed in previous research. Such a finding would represent a next step in unpacking the psychological consequences of perceived endings in everyday life.

## Introduction

Numerous studies examining horse races suggest that individuals tend to show increased risk taking as they are approaching the end of the racing day (McGlothlin, 1956; Ali, 1977; Asch et al., 1982; Metzger, 1985; Kopelman and Minkin, 1991), a phenomenon named as “the last race effect.” More recently, laboratory experiments replicated this phenomenon in other risky decision tasks beyond horse racing (McKenzie et al., 2016; Xing et al., 2018), so this phenomenon has been renamed as “the ending effect” (Xing et al., 2018).

The underlying mechanism of the ending effect received increasing attention from recent literature. With three experiments, McKenzie et al. (2016) showed that increased risk taking in the final round occurred regardless of whether the participants were in the loss or gain domain. More recently, Xing et al. (2018) examined a motivational account which is based on Socioemotional Selectivity Theory (SST, Carstensen et al., 1999; Carstensen, 2006). According to SST, the time perception of an approaching ending leads to motivational shifts from knowledge acquisition to emotional satisfaction. This motivational shift could subsequently lead to changes in cognitive processes (e.g., Mather and Carstensen, 2003, 2005; Isaacowitz et al., 2006a,b) including the decision-making process (e.g., Löckenhoff and Carstensen, 2007).

With four experiments, Xing et al. (2018) showed that the increased motivation to gain extra rewards in order to meet participants’ goal for an emotionally satisfying ending might explain the ending effect. More specifically, compared with non-ending trials, the motivation to gain extra rewards gets stronger than the motivation to avoid losses for the ending trial (Experiment 3 in Xing et al., 2018). However, in Xing et al. (2018), the motivational state was measured after participants had completed all risky decision tasks. Participants’ recalled motivation might be influenced by confounding factors (e.g., the investment outcome of the last round) that is irrelevant with their motivational state *before* the last round. Therefore, it is still unclear whether participants’ motivation toward gaining reward increases *before* the last round.

To fill in this gap of knowledge, the present study directly measured participants’ motivation *before* they worked on the last round of a risky decision task. A visual reaction time task which examines participants’ attention to motivationally significant stimuli (Newman et al., 1993) was adopted. The task requires participants to discriminate between letter and number strings appearing after a warning stimuli. Character strings appear either in the center of the screen or in one of the four corners (see detailed description in the Methods section). Results from previous studies demonstrated that the display of motivationally significant stimuli resulted in slower response times to peripherally located strings (Newman et al., 2010). Therefore, it was hypothesized that, for participants who are approaching the end of their decision task (informed condition), their response time to peripherally located strings would be slower after the presentation of warning stimuli associated with the meaning of gains (gain-words) than those associated with the meaning of losses (loss-words). whereas this pattern would not hold for participants who are not aware of the approaching ending (non-informed condition, reversed or no difference).

## Materials and Methods

### Participants and Design

Seven-two undergraduate students (27 males and 45 females, *M*_age_ = 21.76, *SD*_age_ = 2.62) were recruited, they were randomly assigned to the informed condition (*N* = 36) and the non-informed condition (*N* = 36). Power analyses based on effect size of a previous study ($\eta_{p}^{2}$ = 0.039, see Xing et al., 2018) showed a sample of at least 48 participants was required. All procedures performed in this study conform to the relevant regulatory standards and has been approved by the Psychology Department’s IRB at the university where data was collected.

### Decision Task

We adopted the decision task paradigm used in Xing et al. (2018). Participants completed 11 rounds of a risky decision task. Within each round, participants decided whether to invest or not; if they decided to invest, they were asked to decide the amount of tokens to invest. They were allowed to invest no more than 5 tokens within each round. Then, the experimenter tossed a dice. If the outcome was “1” (1/6 chance), then the participants won; if the outcome was not “1” (5/6 chance), the participants lost. If the participants invested and won, they would receive 7 times of the token(s) they invested; if the participants invested and lost, they would lose the token(s) invested. If the participant decided not to invest, the experimenter still tossed the dice, and the experiment advanced to the next round after the participants knew the outcome of the toss, but no change happened to his/her account.

At the beginning, participants were endowed with 10 tokens and were told that, (1) they would receive in cash for the amount they had remaining after the experiment; (2) if they lost, they could either pay the amount they lost in cash or eliminate their loss by translating a two-page English article into Chinese (similar procedures have been used in previous studies to convince the participants that they could really lose money: (Scholer et al., 2010). In fact, those who were left with fewer than five tokens were paid a minimum of five Chinese Yuan.

### Measurement of Motivationally Significant Stimuli

A visual reaction time task closely modified on a task paradigm used in previous studies (Newman et al., 1993) was used to test individuals’ sensitivity to gain-words and loss-words. A pilot study (*N* = 7) was conducted to select the stimuli (i.e., warning words) to be used in the visual reaction time task: four gain-words, four loss-words, and 12 neutral words. These three types of words did not differ in terms of their familiarity ratings [*M* = 5.83, 5.29, 5.54, *SD* = 0.79, 0.77, 0.76, *F*(2,17) = 0.50, *n.s.*] and number of strokes [*M* = 22.25, 18.5, 20.83, *SD* = 4.65, 4.80, 3.35, *F*(2,17) = 0.96, *n.s.*].

As in the previous study (Newman et al., 1993), the task consisted of a practice block and three test blocks. The practice block contained 24 practice trials and the test block was consisted of 48 test trials. Among the 12 neutral words, eight of them appeared in the practice block, and the other four appeared in the test trial. During each test trial, a warning word first appeared in the middle of the screen for 1000 ms, these words were of three types: gain-words, loss-words, and neutral words. Within each test block, each of the four gain-words, the four loss-words and the four neutral words appeared four times. The presentation order was randomized by E-prime software.

Immediately after the warning word disappeared, a character string (target stimuli) of five letters or five numbers appeared for 2000 ms. The string type (i.e., letter or number) and string composition (i.e., which letters and numbers) within each trial were randomized. In order to establish a dominant attentional set, 75% of the strings were presented in the center of the screen, and the other 25% strings were displayed in the peripheral locations (Newman et al., 1993). Centrally located strings appeared either one line above or one line below the warning stimuli. Peripheral character strings were centered on one of the four corners.

Participants were asked to determine the string type by pressing the pre-designated button as quickly and as accurately as possible, and their reaction times were recorded. After each response, a feedback indicating whether their response was correct or not was displayed for 1000 ms.

### Procedure

Upon arrival at the lab, participants first signed the informed consent form and were given instructions. After two practice trials, participants in the *informed* condition were told that the decision task included 11 rounds, while those in the *non-informed* condition did not know how many decisions they were going to make until they had finished all decisions. Upon finishing the 10th round of the decision task, participants in both conditions worked on the visual reaction time task. Afterwards, all participants continued to finish the last round of the decision task.

## Results

Two participants had a accuracy below 90% in the visual reaction time task, therefore their data was removed, resulting in 35 participants in each condition.

### Decision Data

**FIGURE 1:**
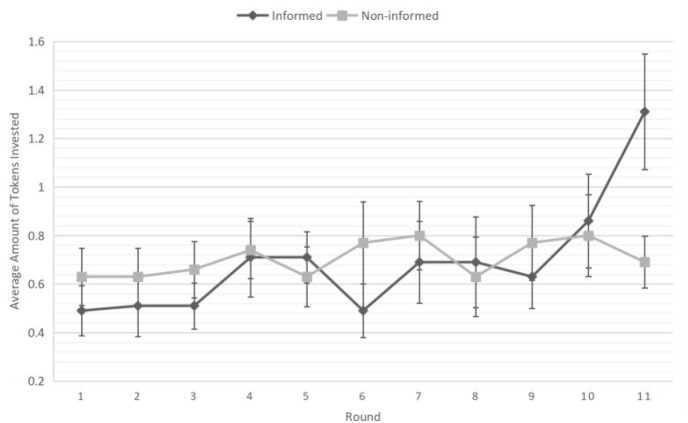
Average investment amount during the 11 rounds of risky decision task. Error bars represent standard errors.

Results of the decision-making data replicated findings in previous studies (McKenzie et al., 2016; Xing et al., 2018). Repeated-measure ANOVA was conducted, with average investment amount in each round as the dependent variables. A significant round X condition interaction effect emerged (see Figure 1), *F*(10,680) = 2.15, *p* = 0.019, $\eta_{p}^{2}$ = 0.031. Participants in the informed condition (*M*_informed_ = 1.31, *SD*_informed_ = 1.41) invested more tokens in the last round than those in the non-informed condition [*M*_non-informed_ = 0.69, *SD*_non-informed_ = 0.63, *F*(1,68) = 5.80, *p* = 0.019, $\eta_{p}^{2}$ = 0.079]. None of the other rounds showed this between group difference, *F*s(1,68) < 1.99, *n.s.*.

### Reaction Time Data

Across the four experiments in Newman et al. (1993), only the data of the first block of trials was analyzed, because the group differences generally disappeared by the second block of trials. Therefore, we also focused on the first block. To better reflect the relative change in reaction time to gain-words and loss-words, we computed a difference score (reaction time index) between target stimuli and neutral stimuli for each target stimuli type and each string location, by subtracting the reaction time to the neutral words from the reaction time to gain-words or loss-words for each of the four within-subject cell (gain-central, gain-peripheral, loss-central, loss-peripheral). A positive score means participants responded slower to the target stimuli (strings) appeared after the gain/loss words than those strings appeared after the neutral warning words. A negative score suggests that participants responded faster to the string appeared after the gain/loss word than those strings appeared after the neutral warning words.

**FIGURE 2:**
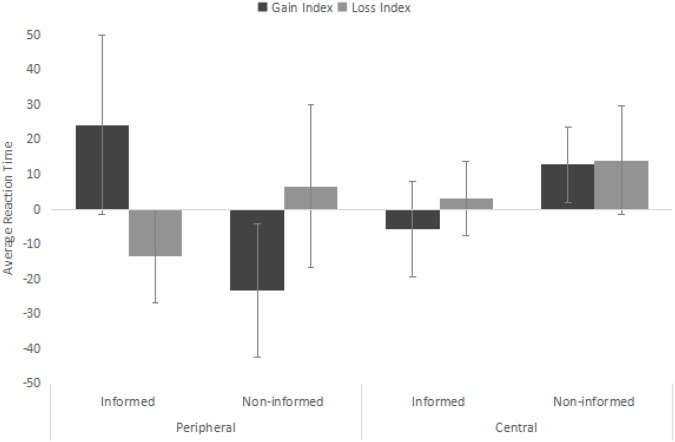
Average reaction time of the eight experimental conditions. Error bars represent standard errors.

Next, a mixed ANOVA was performed with experimental condition as the between-subject variable; the within-subject variables were type of warning words and location of the target stimuli. Reaction time index served as the dependent variable. A significant type X location X condition interaction effect emerged (see Figure 2), *F*(1,68) = 5.16, *p* = 0.026, $\eta_{p}^{2}$ = 0.071. Next, we analyzed this three-way interaction by examining participants’ reaction time to the target stimuli appeared in the central (dominant) location and the peripheral (non-dominant) location separately. For trials in which target stimuli appeared in the peripheral location, the interaction effect between type of warning words and experimental condition emerged, *F*(1,68) = 6.62, *p* = 0.012, $\eta_{p}^{2}$ = 0.090. Simple effect analysis revealed that, in the informed condition, the difference between response times to target stimuli following gain and neutral words (*M*_gain-index_ = 24.14 ms, *SD*_gain-index_ = 105.30 ms) was significantly more positive than the difference between response times to target strings following loss and neutral words (*M*_loss-index_ = -13.47 ms, *SD*_loss-index_ = -73.35 ms), *F*(1,68) = 4.12, *p* = 0.046, $\eta_{p}^{2}$ = 0.060; in contrast, this difference was not significant for participants in the non-informed condition, M_gain-index_ = -23.24 ms, *SD*_gain-index_ = 112.67 ms, *M*_loss-index_ = 6.58 ms, *SD*_loss-index_ = 111.31 ms, *F*(1,68) = 2.59, *n.s.*.

In Newman et al. (1993), only the interference effect of motivationally significant cues was found for processing of the non-dominant response set, yet no evidence was revealed that motivationally significant cues facilitate processing of dominant stimuli. Results from the present study is consistent with Newman et al. (1993). For trials in which target stimuli appeared in the central location, the main effect of type of warning words was not found, *F*(1,68) = 0.51, *n.s.*; neither was there interaction effect between type and experimental condition, *F*(1,68) = 0.62, *n.s.*.

### Mediation Analysis

Next, a mediation analysis was performed to examine whether the effect of perceived ending on the number of tokens invested in the final round could be mediated by the strength of the motivational shift. There was a significant total effect of experimental conditions on the number of tokens participants invested in the final round, such that participants in the informed condition tended to invest more tokens than those in the non-informed condition [*B* = -0.629, *SE* = 0.261, *t*(69) = -2.408, *p* = 0.019, *95% CI*: (-1.149, -0.108)]. Informed conditions produced marginally stronger motivational shift [*B* = -47.379, *SE* = 26.067, *t*(69) = -1.818, *p* = 0.074, *95% CI*: (-99.394, 4.637)] and lower gain index of peripherally located target stimuli (gain index-peripheral) was associated with more tokens invested in the last round [*B* = -0.002, *SE* = 0.001, *t*(69) = -1.585, *p* = 0.118, *95% CI*: (-0.004, 0.000)].

Then, PROCESS was used to calculate the indirect effect of experimental conditions on the number of tokens participants invested in the final round through gain index-peripheral. The indirect effect was 0.090, *SE* = 0.071, *95% CI*: [0.0001, 0.3009], with 5000 bootstrap samples, leaving a significant direct effect of -0.7188, *SE* = 0.264, *t*(69) = -2.72, *p* = 0.008, *95% CI*: [-1.2465, -0.1911]. The proportion of mediated effect was -0.143, which indicated that this pathway accounts for about 14.3% of the effect.

## Discussion

The present study replicated the ending effect suggested in previous research (McGlothlin, 1956; Ali, 1977; Asch et al., 1982; McKenzie et al., 2016; Xing et al., 2018): individuals who knew they were working on the last round of a risky decision task showed increased risk taking in the last round. More importantly, this study compared the motivational priorities between participants who believed they were working on the ending trial and those who thought they were working on non-ending trials. Through a visual reaction time task established in previous study to test motivationally significant stimuli (Newman et al., 1993), we examined participants’ motivation toward gaining rewards and their motivation toward avoiding losses, immediately before they worked on the last round of a risky decision task. The results revealed that perceived endings lead participants get more concerned with gains than losses, as they respond slower to gain-words and faster to loss-words that appeared at the peripheral location. In the non-informed condition, descriptive data of reaction time revealed the reversed pattern, though the difference between participants’ response times to gain-words and loss-words was not statistically significant. More importantly, the effect of perceived ending on individuals’ increased risk taking in the last round was mediated by the motivational effect. This finding provides additional support for the motivational account of the ending effect proposed in recent research (Xing et al., 2018). Future studies might further explore individuals’ motivational priority toward gains and losses for non-ending trials.

This study, together with the four experiments reported in Newman et al. (1993), consistently suggest that the effect of motivationally significant stimuli only emerges in the peripheral, non-dominant display. These results provide consistent support for the notion that motivationally significant stimuli interfere with individuals’ ability to process information presented in the peripheral, non-dominant location, and such stimuli does not facilitate processing of information presented in the central, dominant location.

One limitation of the present findings is that the effect sizes are relatively small. Therefore, future replication studies are needed to confirm the findings from the present work. Another limitation of this study is that we only used the implicit measurement of individuals’ motivation. This was because we were concerned using self-report questions (explicit measurement) to measure motivation might affect participants’ decision in the final round. Future studies would benefit by exploring other methods to measure individuals’ motivational state and comparing results from different methodologies.

The present study contributes to SST in two ways. First, it provides direct evidence to support the idea that perceived endings lead to motivational shift. Second, previous studies examining the prediction of SST have mainly focused on endings of major life events; the present finding together with previous studies examining the ending effect (McKenzie et al., 2016; Xing et al., 2018) provide consistent evidence that everyday endings also influence individuals’ psychological processes and behaviors. Given that everyday endings are far more common than endings of major life events, systematic investigation of the psychological consequences of everyday endings is a promising direction for future research.

## Ethics Statement

All procedures performed in studies involving human participants were in accordance with the ethical standards of the institutional and/or national research committee and with the 1964 Helsinki declaration and its later amendments or comparable ethical standards. Informed consent was obtained from all individual participants included in the study.

## Author Contributions

CX designed the experiments and drafted the manuscript. YM performed the data analyses and drafted the manuscript. DI drafted the manuscript. YS recruited participants and collected data. JC wrote the Eprime program and recruited participants.

## Conflict of Interest Statement

The authors declare that the research was conducted in the absence of any commercial or financial relationships that could be construed as a potential conflict of interest.
